# A use-dependent increase in release sites drives facilitation at calretinin-deficient cerebellar parallel-fiber synapses

**DOI:** 10.3389/fncel.2015.00027

**Published:** 2015-02-03

**Authors:** Simone Brachtendorf, Jens Eilers, Hartmut Schmidt

**Affiliations:** Medical Faculty, Carl-Ludwig-Institute for Physiology, University of LeipzigLeipzig, Germany

**Keywords:** short-term plasticity, calretinin, paired-pulse facilitation, paired recordings, granule cells, ready-releasable pool

## Abstract

Endogenous Ca^2+^-binding proteins affect synaptic transmitter release and short-term plasticity (STP) by buffering presynaptic Ca^2+^ signals. At parallel-fiber (PF)-to-Purkinje neuron (PN) synapses in the cerebellar cortex loss of calretinin (CR), the major buffer at PF terminals, results in increased presynaptic Ca^2+^ transients and an almost doubling of the initial vesicular releases probability (*p*_r_). Surprisingly, however, it has been reported that loss of CR from PF synapses does not alter paired-pulse facilitation (PPF), while it affects presynaptic Ca^2+^ signals as well as *p*_r_. Here, we addressed this puzzling observation by analyzing the frequency- and Ca^2+^-dependence of PPF at unitary PF-to-PN synapses of wild-type (WT) and CR-deficient (CR^−/−^) mice using paired recordings and computer simulations. Our analysis revealed that PPF in CR^−/−^ is indeed smaller than in the WT, to a degree, however, that indicates that rapid vesicle replenishment and recruitment of additional release sites dominate the synaptic efficacy of the second response. These Ca^2+^-driven processes operate more effectively in the absence of CR, thereby, explaining the preservation of robust PPF in the mutants.

## Introduction

Ca^2+^ regulates use-dependent presynaptic short-term plasticity (STP) by controlling the initial vesicular releases probability (*p*_r_), the facilitation status of the release apparatus, and by regulating the size and restoration of vesicle pools (Zucker and Regehr, 2002; Regehr, 2012). Endogenous Ca^2+^ buffers (CaBs) are important regulators of presynaptic Ca^2+^ signals (Eggermann et al., 2012; Schmidt, 2012). Due to their diffusional mobility (Schmidt et al., 2003, 2005; Arendt et al., 2013), they get close to the site of Ca^2+^ entry and buffer the release triggering Ca^2+^ signal even if the diffusional distance between Ca^2+^ channel and release sensor is a few tens of nanometers only (Eggermann and Jonas, 2012; Bornschein et al., 2013; Schmidt et al., 2013). Well-known examples of at least partly mobile neuronal CaBs involved in the regulation of STP include parvalbumin (PV; Caillard et al., 2000), and calbindin-D28k (CB; Blatow et al., 2003).

Calretinin (CR) is an endogenous CaB closely related to CB. Four of its binding sites bind Ca^2+^ in a cooperative manner with partly rapid kinetics (Faas et al., 2007). Thus, similar to loss of CB (Bornschein et al., 2013), loss of CR results in significantly increased synaptic Ca^2+^ transients, reduced initial synaptic failure rate (F1), and increased *p*_r_ (Schmidt et al., 2013). Surprisingly, however, at the same synapses, the cerebellar parallel-fiber (PF)-to-Purkinje neuron (PN) synapses, which express CR as their major buffer, loss of CR has been reported not to be associated with significant alterations in paired-pulse facilitation (PPF; Schiffmann et al., 1999). In order to resolve this discrepancy, we analyzed PPF at unitary PF-to-PN connections in recordings from pairs of connected granule cells (GCs) and PNs in wild-type (WT) and CR deficient (CR^−/−^) mice over a broad range of frequencies (5–1000 ms) and extracellular Ca^2+^ concentrations (1–10 mM). Contrary to the previous report (Schiffmann et al., 1999), we found that loss of CR resulted in a significant reduction in PPF. This reduction, however, was less prominent than would have been expected for the high *p*_r_ at CR^−/−^ synapses. The fraction of synaptic failures to the second stimulus (F2) was significantly reduced in the mutants, suggesting that a rapid recovery of the releasable vesicle pool (RP) may compensate for its depletion during the first stimulus. Experimentally constrained computer simulations combined with an analysis of successes and failures, and multiple probability fluctuation analysis (MPFA) suggest the involvement of a Ca^2+^-driven mechanism in PPF (Millar et al., 2005; Sakaba, 2008; Valera et al., 2012), which restores and overfills the RP more effectively in CR^−/−^ than in WT, thereby, essentially preserving PPF in the mutants.

## Materials and methods

### Slice preparation

Horizontal cerebellar slices (300 μm thick) were prepared (HM 650 V; Microm, Walldorf, Germany) from the vermis region of 21–24-day-old C57BL/6 and CR^−/−^ mice (Schiffmann et al., 1999). The animals were decapitated following anesthesia with isoflurane (Curamed, Karlsruhe, Germany) and the cerebella were rapidly removed and placed in cooled (0–4°C) artificial cerebrospinal fluid (ACSF) containing (in mM): 125 NaCl, 2.5 KCl, 1.25 NaH_2_PO_4,_ 26 NaHCO_3,_ 1 MgCl_2_, 2 CaCl_2_ and 20 glucose, bubbled with 95% O_2_ and 5% CO_2_ (pH 7.3–7.4 at 20–22°C). Slices were kept for 30 min. at 35°C prior to the experiments. Unless stated otherwise, all chemicals were obtained from Sigma-Aldrich, Seelze, Germany.

### Electrophysiology

Patch pipettes were prepared from borosilicate glass (Hilgenberg, Malsfeld, Germany) with a PC-10 puller (Narishige, Tokyo, Japan) and had final resistances of 4–5 MΩ or 10–11 MΩ when filled with intracellular solution for recordings from PNs or GCs, respectively. The intracellular solution contained (in mM): 150 K-gluconate, 10 NaCl, 3 Mg-ATP, 0.3 GTP, 10 HEPES and 50 μM EGTA dissolved in purified water (Sigma-Aldrich, Seelze, Germany).The pH was adjusted to 7.3 with KOH. Slices were transferred to the bath chamber perfused continuously at 3 ml/min. with ACSF containing 10 μM of the GABA_a_-receptor blocker gabazine (SR-95531). Experiments were carried out at room temperature. Cells were visualized with an upright microscope (BX50WI, Olympus, Lambrecht, Germany) equipped with a 60×/0.9 NA water immersion objective (Olympus). Somatic whole-cell patch-clamp recordings from PNs were obtained using an EPC10**-**2 amplifier (HEKA, Lambrecht, Germany) and “Patchmaster 2.6” software. The liquid junction potential (15 mV) was corrected for. During the experiments, the series resistance and leak current were monitored continuously and experiments were rejected if either the series resistance exceeded 30 MΩ, deviated by more than 15% from its initial value, or the leak current fell below −350 pA.

Stimulation of individual GCs was achieved in the loose-cell attached configuration in the voltage-clamp mode by using the current response of the amplifier to positive voltage steps (Perkins, 2006) and borosilicate patch-pipettes (10–11 MΩ) filled with intracellular solution. GCs connected to the patched PN were located by briefly puffing the potassium containing intracellular solution to the GC layer at a distance of > 100 μm laterally from the PN. If current responses > 20 pA were recorded from the PN during puff-application, GCs in this region were tested sequentially by using electrical stimulation (stimulus duration: 500 ms; 10 repeats) in the loose-patch configuration with reuse of the pipette. Upon identification of a connected GC, stimulations at different interstimulus intervals (ISI: 5–100 ms) were performed with an inter-sweep interval of 5 s. Excitatory postsynaptic currents (EPSCs) were recorded in PNs at a holding potential of −70 mV to −80 mV, filtered at 5 kHz and sampled at 10 kHz. The stimulus duration was ≤2 ms and the stimulus amplitude was adjusted to induce a single action current only, in order to avoid stimulation of neighboring cells or fibers (Schmidt et al., 2013).

All experiments were carried out in accordance with institutional guidelines for animal experiments, and were approved by the state directorate of Saxony, Germany.

### Analysis and statistics

EPSCs were analyzed with “Patchmaster 2.6” software and custom written routines in Igor Pro 6.21 (Wavematrics, Lake Oswego, Oregon). paired-pulse ratios (PPRs) were calculated by dividing the average of the second EPSC amplitude of a given ISI by the average of the first EPSC amplitude of all ISIs at a given synapse. Amplitudes were determined by fitting a product of two exponential functions to the baseline-subtracted currents, which allowed for independent adjustment of the time constants of the rising and falling phases. The amplitude of the second EPSC was determined as the difference between its peak and the decay of the first EPSC, thereby eliminating the effects of electrical summation in particular at short ISIs. EPSCs were classified as failures if their amplitude was < 5 pA i.e., 2-fold root mean square (RMS) noise. It cannot be excluded that some EPSCs with smaller amplitude remained undetected.

Quantal synaptic parameters were determined from EPSC amplitudes recorded at an ISI of 10 ms at different [Ca^2+^]_e_ (1, 2 and 10 mM, ≥50 repetitions per concentration) and a 5 s interval between paired stimulations. In indicated experiments 5 mM of the competitive low-affinity AMPA receptor antagonist γ-D-glutamylglycine (γ-DGG) was added to the bath to relieve postsynaptic receptor saturation.

For MPFA, which corrected for non-uniform quantal size (Clements and Silver, 2000; Scheuss et al., 2002), noise-corrected variances (*σ*^2^) were plotted against mean EPSC amplitudes (*I*) and fitted by a parabola of the form:

(1) $$\sigma^{2}=Iq-\frac{I^{2}}{N}\left(1+CV_{II}^{2}\right)+qICV_{I}^{2}$$

with q being the quantal size, N a binominal parameter indicating the number of release sites or releasable vesicles, and *CV_I_* and *CV_II_* the coefficients of intra- and inter-site quantal variability, assumed to be 0.3 (Valera et al., 2012; Schmidt et al., 2013). Non-integer values for N were allowed to indicate the uncertainty of the fit (for further detail see Hallermann et al., 2010; Schmidt et al., 2013). MPFA is well suited for unitary connections (Clements and Silver, 2000) and has previously been shown to be applicable at the unitary PF to PN synapses (e.g., Sims and Hartell, 2006; Valera et al., 2012; Schmidt et al., 2013).

Unless stated otherwise, data are presented as mean and ± SE. Parametric statistical tests were performed if data were normally distributed (Shapiro-test) with equal variance and if the number of data points was sufficiently large (as indicated by the power of the test); non-parametric tests were used otherwise or in addition, as indicated. Statistical significance was tested for either with the *t*-test (two groups, normally distributed), the Mann-Whitney rank sum test (two groups, non-normally distributed) or with parametric (more than two groups, normally distributed) or nonparametric (more than two groups, either non-normally distributed or normally distributed and low n; *post hoc* testing with Dunn’s method) ANOVA designs, using Sigma Plot 11.0 software (Erkrath, Germany).

### Simulation of Ca^2+^ dynamics and facilitated transmitter release

The reaction schemes of a kinetic model were transformed into the corresponding ordinary differential equations and numerically solved using Mathematica 10.0 (Wolfram Research). The model (Schmidt et al., 2013) included a Gaussian-shaped Ca^2+^ influx, buffering of Ca^2+^ by ATP, Calmodulin, and cooperative CR (Faas et al., 2007), Ca^2+^ extrusion, diffusion of all species and Ca^2+^-dependent vesicle fusion and replenishment (Millar et al., 2005; Sakaba, 2008). Resting Ca^2+^ was set to 45 nM. Release rates were obtained by differentiation of the fused state. PPRs were calculated from the *p*_r_ ratios, obtained by integration of the release rates. The model was adjusted to yield an initial *p*_r_ of ~0.25 and ~0.41 in WT and CR^−/−^, respectively (Schmidt et al., 2013), and a PPR at 10 ms ISI consistent with the experiments.

Parameters of the release and replenishment model were similar to previously published values (Millar et al., 2005; Sakaba, 2008). The release sensor (V) was modeled with a binding rate *k*_on_ = 1.2*10^8^ M^−1^s^−1^, unbinding rate *k*_off_ = 1000 or 3500 s^−1^, cooperativity *b* = 0.3, and release rate *g* = 10000 s^−1^. The replenishment part (R_0_, R_1_) was simulated with forward and backward rate constants of Ca^2+^-dependent priming and unpriming of *k*_prim_ = 3.5*10^8^ or 1.8*10^8^ M^−1^s^−1^ and *k*_unprim_ = 500 s^−1^, respectively, followed by Ca^2+^-independent rates for forward and backward transition into or out of the RP with *k*_t+_ = 500 s^−1^ and *k*_t-_ = 50 s^−1^, respectively. For simplicity, release and replenishment were driven by the same local Ca^2+^ signal (Millar et al., 2005). Model settings were identical for WT and CR^−/−^ and the mutant was simulated by only removing CR from the model.

## Results

### Identification of GC-to-PN pairs

**Figure 1:**
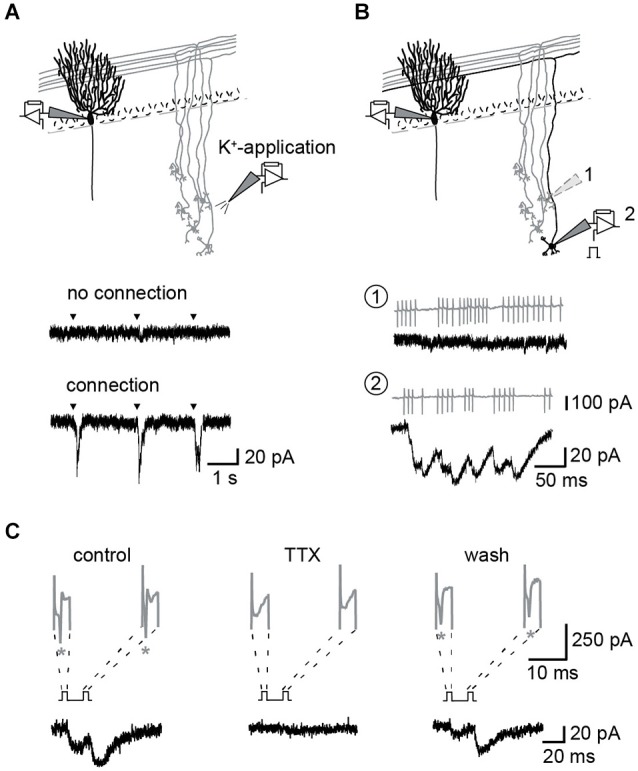
**Identification of unitary GC–PN connections. (A,B)** Schemes of experimental approaches. **(A)** Top: PNs were held in the whole-cell configuration. GCs were stimulated via puffs of K^+^-containing pipette solution applied to the granule cell layer (GCL). Example recordings from a PN during K^+^-application to different regions of the GCL are shown below. Middle: no activation of connected GCs; bottom: inward currents recorded in response to activation of connected GCs. Triangles indicate times of K^+^-application to the GLC. **(B)** Top: Single GCs were stimulated in the loose-cell attached configuration within the region that responded to K^+^-puffs. *Bottom*: Example recording from a not connected (1) and a connected (2) GC-PN pair. Action currents (ACs) (gray) elicited in the GC and the corresponding postsynaptic response (black). **(C)** Presynaptic ACs were reversibly inhibited by bath application of 1 μM TTX. Currents from GC (gray; stars denote ACs; initial peaks are capacitive currents) and the corresponding current traces (black) recorded from the connected PN.

The GC to PN connectivity is rather low in slice preparations (Isope and Barbour, 2002) and we used the following procedure to establish paired recordings (Figure 1): After the whole-cell configuration had been established on a PN, potassium containing pipette solution was puffed from a second patch-pipette to regions of the GC layer at a distance of > 100 μm from the soma of the PN. This distance was chosen to ensure potassium-mediated activation of PF synapses rather than synapses formed by the ascending axon of the GC (Isope and Barbour, 2002). If cells or fibers connecting to the PN were present in this region, EPSCs were apparent in the recording from the PN soma (Figure 1A). Subsequently, individual GCs in this region were activated by electrical stimulation of their somata in the loose-cell configuration (Perkins, 2006) making repeated use of the same patch-pipette (Figure 1B). After identification of a GC-PN pair, tetrodotoxin (TTX) sensitive action currents (ACs) were elicited in the GC by brief current pulse (< 2 ms) at different ISI and evoked EPSCs recorded from the PN (Figure 1C). Assuming reliable propagation of the action potential (AP; Isope and Barbour, 2002), stimulation and release failures could be clearly distinguished in these recordings (cf. Schmidt et al., 2013).

### Reduced failure rates in the second response contributes to PPF

**Figure 2:**
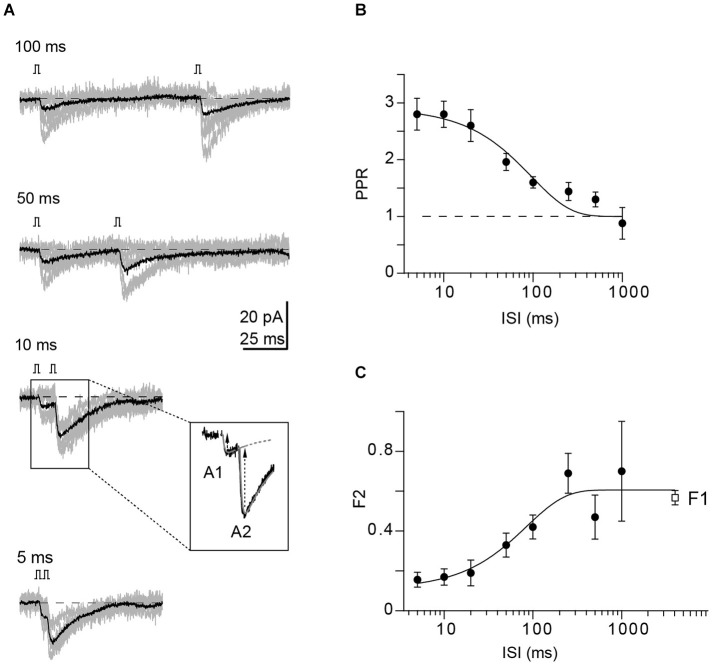
**Properties of connected GC-PN pairs in the WT. (A)** Example of a paired-pulse experiment at denoted ISI from a unitary GC-PN connection (individual traces in gray; average, including failures, in black). Inset: EPSC amplitudes (black arrows) were determined by fitting products of two exponentials (dashed black lines) to the currents, allowing for subtracting the decay of the first EPSC (A1) from the peak of the second EPSC (A2). **(B)** PPRs plotted against the indicated ISI (mean ± SE, *n* = 12). The solid line represents an exponential fit to the data (*τ* = 96 ms; *χ*^2^ = 1.435). **(C)** Fraction of synaptic failures in the second response (F2) plotted vs. the indicated ISI (mean ± SE, *n* = 12). The fraction of synaptic failure in the first response (F1 = 0.57 ± 0.04, *n* = 16) is plotted at the interval between successive recordings (5 s). Solid line represents exponential fit to the data (*τ* = 85 ms; *χ*^2^ = 0.621).

Following the establishment of a recording from a GC-PN pair connected via a PF synapse, we recorded the frequency dependence of PPF at ISIs ranging from 5 to 1000 ms (Figure 2A). PPF was maximal (2.8; *n* = 12 pairs) at 5–10 ms ISI. It declined to unity with a time constant (*τ*) of 96 ms (Figure 2B). The rate of synaptic failures in the second response (F2) was lowest at short ISIs and increased with the interval between stimuli until it reached the value of the first responses (F1) at ~300 ms (Figure 2C).

Provided that the quantal size (q) remains unaltered between first and second pulse, the EPSC2/EPSC1 PPR is given by (N2 *p*_r2_)/(N1 *p*_r_), with N1 and N2 indicating the number of available release sites or releasable vesicles at the first and second activation, respectively. Thus, F2 < F1 and PPF result either from increasing *p*_r2_ or N2 or both. N2 is reduced initially by vesicles released during the first pulse but may be replenished by a fraction R between pulses, i.e., N2 = N1 − (N1 *p*_r_) + R (N1 *p*_r_). The above results have interesting implications for the mechanisms of PPF at PF synapses: The PPR of 2.8 at short ISI is close to the theoretical maximum of 3 in the absence of vesicle replenishment [*p*_r2_ = 1; PPR_max_ = 1/$p_{r}^{*}$(1−*p*_r_)], using *p*_r_ of 0.25 for WT PF synapses (Sims and Hartell, 2005; Valera et al., 2012; Schmidt et al., 2013). However, our failure analysis showed that at ISI of 5 and 10 ms F2 was 0.16 ± 0.04 and 0.17 ± 0.04, respectively, demonstrating that *p*_r2_ has not reached a value of one but is ≤ ~0.84 [F = (1−*p*_r_)*^N^*]. Thus, rapid replenishment of N (Crowley et al., 2007) and/or an increase in N (Sakaba, 2008; Valera et al., 2012) may be involved in PPF. Assuming that this process is Ca^2+^-dependent (Millar et al., 2005; Sakaba, 2008), it could provide a mechanistic explanation for PPF in CR^−/−^ since in the absence of this endogenous Ca^2+^ buffer from the synapse it could operate more effectively.

### PPF at individual CR-deficient PF synapses

**Figure 3:**
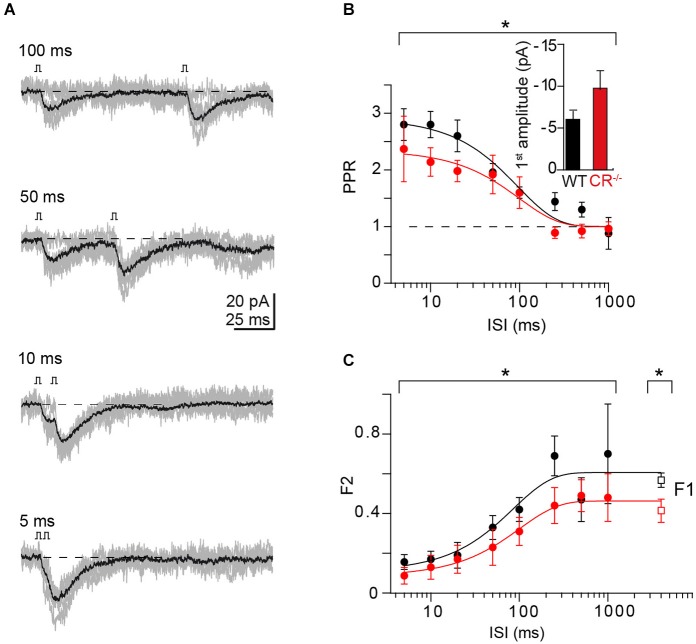
**PPF at unitary CR^−/−^ synapses. (A)** Example of paired-pulse stimulation at indicated ISI from a unitary CR^−/−^ GC-PN connection (individual traces in gray; average, including failures, in black). **(B)** Comparison of PPRs vs. ISI in WT (cf. Figure 2B) and CR^−/−^ (red, mean ± SE, *n* = 6). Solid lines represent exponential fits to the data (CR^−/−^: *τ* = 100 ms; *χ*^2^ = 0.73). PPF was significantly reduced in CR^−/−^ compared to WT (PPR = 2.4 ± 0.6 at 5 ms and 2.1 ± 0.3 at 10 ms; *P = 0.038 with a two-way ANOVA for the complete frequency range; *P* < 0.01 with Kruskal-Wallis (K-W) ANOVA). Inset: Average first EPSC amplitude (including failures) in WT and CR KO (mean ± SE, *n* = 16 and 11, respectively; *P* = 0.059, *t*-test). **(C)** Comparison of the frequency dependence of synaptic failures in the second response (F2) in WT (black; cf. Figure 2C) and CR^−/−^ (red; mean ± SE, *n* = 6; *P = 0.022 two-way ANOVA, *P* < 0.01 with K-W ANOVA) terminals. The initial failure rate (F1) was determined at the interval between successive recordings (CR^−/−^: *n* = 11, *P = 0.027, *t*-test). Solid lines represent exponential fits to the data (CR^−/−^: *τ* = 101 ms; *χ*^2^ = 0.156).

To probe whether Ca^2+^-dependent replenishment and/or increase in N (either by overfilling (Sakaba, 2008) or by recruitment of additional, reluctant release sites (Valera et al., 2012)) could explain the unexpected observation that PPF in CR^−/−^ is not different from WT synapses (Schiffmann et al., 1999), we analyzed the frequency-dependence of PPF in recordings from pairs of connected GCs and PNs in CR^−/−^ mice (*n* = 6 pairs; Figure 3A), using the above described procedure. In contrast to the previous report of unaltered PPF in CR^−/−^ obtained with fiber tract stimulations (Schiffmann et al., 1999), in our paired recordings the magnitude of PPF was moderately but significantly reduced in CR^−/−^ compared to WT in particular at ISI ≤20 ms (Figure 3B; PPR = 2.37 ± 0.58 at 5 ms and 2.14 ± 0.25 at 10 ms; *P* = 0.038). PPR showed a frequency dependence almost identical to the WT, dropping with a time constant of 100 ms to unity. Consistent with a reduced PPF (and an increased *p*_r_) F1 was significantly reduced in CR^−/−^ compared to the WT (0.41 ± 0.06 and 0.57 ± 0.04 for CR^−/−^ and WT, respectively, *P* = 0.027).

PPF in the mutants was still unexpectedly large. Using the above calculations and a *p*_r_ of 0.41 (Schmidt et al., 2013) the theoretical PPR_max_ (for *p*_r2_ of 1) is ~1.4 in the absence of replenishment. With full replenishment (N2 = N1 => PPR = *p*_r2_ /*p*_r_) PPR_max_ would be ~2.4, i.e., the measured PPR substantially exceeds the theoretical PPR_max_ without replenishment and at an ISI of 5 ms is close to PPR_max_ with 100% replenishment. F2 in the mutants was significantly reduced compared to WT (Figure 3C; *P* = 0.022) and, as in the WT, reached the F1 value at ISI ~300 ms. Yet, it did not drop to 0 even at an ISI of 5 ms (0.09 ± 0.04), i.e., also in the mutants *p*_r2_ did not reach a value of one but rather is ≤ ~0.91. This suggests that even a full replenishment of N between pulses is not sufficient to account for the measured PPF in CR^−/−^. In addition, these data suggest that the control of the size of N is dependent on Ca^2+^, since it operated more effectively in the absence of CR.

### PPF at high extracellular Ca^2+^ concentrations exceeds theoretical maxima

**Figure 4:**
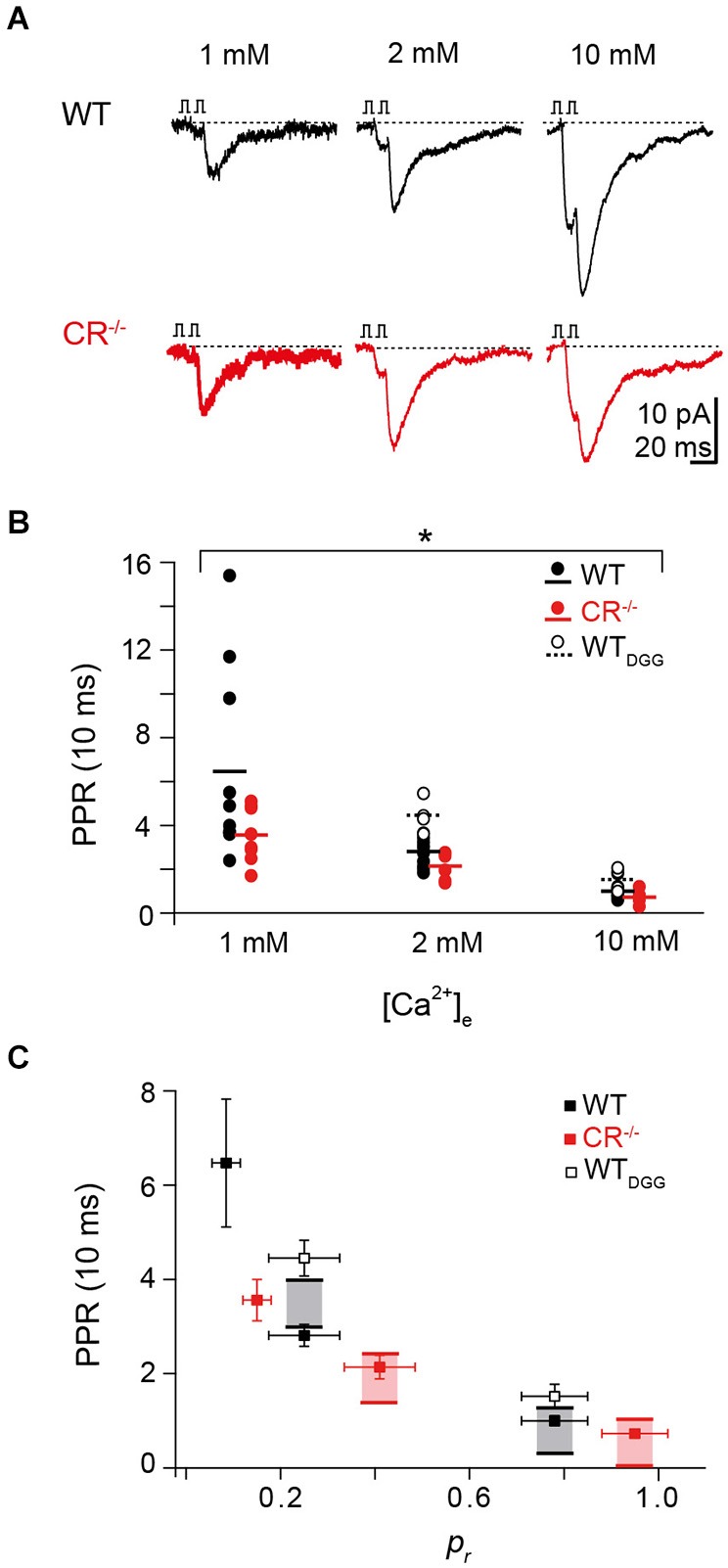
**Rapid replenishment and overfilling contribute to PPF. (A)** Examples of EPSCs recorded at an ISI of 10 ms at different extracellular Ca^2+^-concentrations from a representative WT (black) and CR^−/−^ (red) unitary connection. **(B)** PPR vs. Ca^2+^-concentration from individual pairs at an ISI of 10 ms for WT (black; solid circles, solid line indicates the average; *n* = 10), CR^−/−^ (red; *n* = 8), and WT in the presence of γ-DGG (5 mM, WT_DGG;_ open circles; dashed line indicates average; *n* = 4). For all [Ca^2+^]_e_ concentrations tested, PPR in the mutant was significantly smaller than in WT (*P < 0.01, K-W ANOVA). **(C)** Plot of PPR vs. *p_r_* for all [Ca^2+^]_e_ revealing a decline in PPR with increasing *p*_r_ in WT and CR^−/−^ (PPR: mean ± SE (cf. Panel **B**), *p_r_*: median ± SE; WT (black): *n* = 10; CR^−/−^ (red): *n* = 8). Shaded areas indicate the maximum theoretical values of PPR (PPR_max_ for *p*_r2_ = 1) in presence (top line) and in absence (bottom line) of full vesicle replenishment. PPR values at high *p_r_* are close to the theoretical PPR_max_ with full vesicle replenishment for mutant and WT. PPR values determined in the presence of γ-DGG (open squares, WT_DGG_: *n* = 4) exceed the theoretical PPR_max_ with full vesicle replenishment.

To further test the hypothesis that a Ca^2+^-dependent increase in N contributes to PPF, we measured PPF in paired recordings at WT and CR^−/−^ unitary connections at different extracellular Ca^2+^ concentrations ([Ca^2+^]_e_) of 1, 2, and 10 mM at an ISI of 10 ms (Figure 4A). Alterations in [Ca^2+^]_e_ alter *p*_r_ but may also affect the replenishment/recruitment of N, if Ca^2+^ dependent. We found that for all [Ca^2+^]_e_ concentrations tested, PPR in the mutant was significantly smaller than in WT (Figure 4B; *P* < 0.01). PPF was strongest at 1 mM [Ca^2+^]_e_ with an average PPR of 6.47 ± 1.36 in the WT and 3.56 ± 0.44 in CR^−/−^. The PPR dropped with increasing [Ca^2+^]_e_ being only 1.00 ± 0.11 and 0.73 ± 0.08 in WT and CR^−/−^ in 10 mM [Ca^2+^]_e_, respectively.

It has been shown previously that single EPSC amplitudes at PF synapses are not significantly affected by postsynaptic receptor saturation at high [Ca^2+^]_e_ (Valera et al., 2012; Schmidt et al., 2013). Yet, the PPR analysis may underestimate the magnitude of presynaptic facilitation due to saturation of postsynaptic receptors in the second response. We therefore recorded PPRs in WT in the presence of the competitive low-affinity AMPA receptor antagonist γ-DGG (*n* = 4) which relieves receptor saturation (Foster et al., 2005). Under these conditions, PPR at 2 mM and 10 mM [Ca^2+^]_e_ increased to 4.45 ± 0.38 and 1.52 ± 0.26, respectively (Figure 4B), indicating that receptor saturation indeed led to an underestimation of the real magnitude of presynaptic facilitation.

Plotting the PPR values for each [Ca^2+^]_e_ against the previously quantified *p*_r_ values (Schmidt et al., 2013) revealed the expected dependency of PPF on *p*_r_ in both genotypes (Figure 4C). Inclusion of the theoretical PPR_max_ values in the absence of replenishment (lower boundary of the shaded areas in Figure 4C) showed a clear discrepancy to the measured PPR at 2 and 10 mM [Ca^2+^]_e_ for the mutant and in 10 mM [Ca^2+^]_e_ also for the WT, with the experimental values being larger than the theoretical PPR_max_ value. These data underline that a rapid vesicle pool restoration or recruitment mechanism is likely to contribute to PPF. Remarkably, PPR values determined in the presence of γ-DGG substantially exceeded even the theoretical PPR_max_ with full replenishment, demonstrating that replenishment and increased *p*_r2_ alone are not sufficient to explain PPR, i.e., an increase in N is required to explain the experimentally observed PPF value. In addition, these data further substantiate the above notion that the mechanism controlling the size of N is Ca^2+^-dependent since it was influenced by alterations in [Ca^2+^]_e_ and operated more effectively in the absence of the endogenous Ca^2+^ buffer CR.

### Use dependent increment in N can account for PPF

In order to further test the hypothesis that replenishment and increasing N contribute to the only moderate reduction of PPF in CR^−/−^, we analyzed a model of release and replenishment that allows for increasing N2 above N1. This model was previously shown to reproduce central characteristics of phasic and tonic synapses (Millar et al., 2005; Sakaba, 2008). In this model, release is triggered via a cooperative Ca^2+^-driven five-site sensor from a population of releasable vesicles. These vesicles become replenished in two steps with the first step being Ca^2+^ dependent.

**Figure 5:**
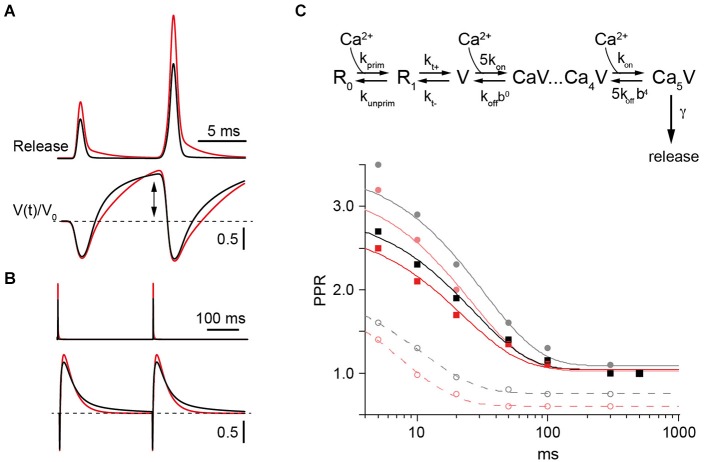
**Model of PPF in WT and CR^−/−^ synapses. (A)** Upper: Simulated transmitter release rates during a pair of synaptic activations at an ISI of 10 ms in WT (black) or CR^−/−^ synapses (red) normalized to the first release process in the WT. Lower: Temporal variation of Ca^2+^-free release sensor sites (V(t)) normalized to their value prior to the first stimulus (V_0_) during 100 Hz activation in WT (black) or KO synapses (red). Note the increase (arrow) above V_0_ (dashed line) between pulses. **(B)** Same as in **(A)** but for two activations with an ISI of 300 ms. **(C)** Upper: Scheme of the model which includes a release sensor with 5 Ca^2+^ binding sites (V) and a two-step replenishment (R_0_, R_1_) with the first step being Ca^2+^ dependent and the second step indirectly Ca^2+^ dependent (Millar et al., 2005; Sakaba, 2008). Lower: Paired pulse ratios (PPRs) calculated as the ratio of release probabilities between the second and the first pulse plotted against the ISI. Lines represent exponential fits. Curves for the WT are in gray and black and those for the CR^−/−^ in red. Solid gray and light red curves represent simulations with *k*_off_ set to 1000 s^−1^ (Millar et al., 2005). The corresponding PPRs result from the combined action of Ca^2+^ remaining bound to the release sensor (active Ca^2+^) between stimuli at small ISI (dashed lines) and overfilling of the pool V (cf. **(A)**). Increasing *k*_off_ to 3500 s^−1^ eliminated facilitation due to active Ca^2+^ and resulted in the black and red curves for the frequency dependence of PPR in WT and KO, respectively. Irrespective of model details only slightly decreased facilitation is predicted for the KO in comparison to the WT.

In our simulations the release-triggering Ca^2+^ signals in WT and CR^−/−^ were adjusted according to previously measured Ca^2+^ transients in PF boutons and corresponding estimates of the Ca^2+^ dynamics at the release sites (Schmidt et al., 2013). For simplicity, Ca^2+^ -dependent replenishment was driven by the same local Ca^2+^ signal (Millar et al., 2005). The Ca^2+^-dependent replenishment steps allow for a transient, several hundred ms long increment in N, corresponding to an overfilling or recruitment process (Figures 5A,B; Millar et al., 2005; Sakaba, 2008). The major endogenous CaB used was CR, and the two genotypes were simulated by either including (WT) or removing (CR^−/−^) CR from the simulations without changing other parameters.

Pairs of release processes were modeled at ISI of 5–500 ms. PPRs were calculated from the ratio of the time-integrals of the release rates. Plotting the resulting PPRs against the corresponding ISI showed a simulated frequency dependence of facilitation that was in good accordance to the experimental values in both genotypes. In particular, a slightly reduced PPF in CR^−/−^ compared to the WT was obtained (Figure 5C). When the replenishment steps were excluded from the simulations a residual facilitation remained at short ISI of ≤20 ms (dashed lines in Figure 5C). This resulted from the release sensor remaining facilitated for short intervals determined by its Ca^2+^ unbinding rate (“active Ca^2+^”, Katz and Miledi, 1968; Millar et al., 2005; Bornschein et al., 2013). The magnitude of PPF caused by the active Ca^2+^, however, was too small to account for the experimental values as expected from the uncompensated depletion during the first release process, indicating that the transient increase in N is a major determinant of PPF (solid lines in Figure 5C).

**Figure 6:**
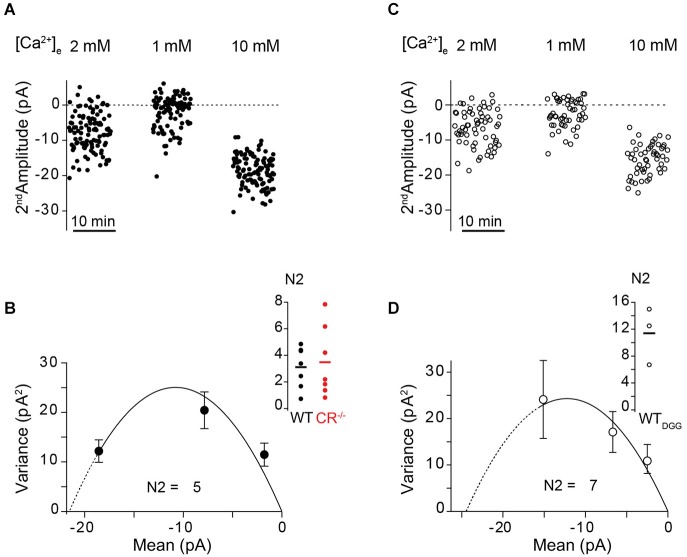
**Use dependent increase in N quantified by MPFA. (A,C)** Fluctuation analysis of second EPSC amplitudes recorded at an ISI of 10 ms at the indicated [Ca^2+^]_e_ from an unitary WT GC-PN connection in the absence (**(A)**, closed circles) and in the presence of γ-DGG (**(C)**, open circles), respectively. **(B,D)** Corresponding variance–mean relationships of the noise-corrected second EPSC amplitudes. Error bars show the variance of the variance. The solid lines represent the parabolic MPFA fit (**(B)**, *χ*^2^ = 3.709; **(D)**, *χ*^2^ = 0.665), which yielded the binominal parameter N for the second pulse (N2). Line dashing indicates the region over which the fit has been extrapolated. Inset: Summary of the estimated N2 values in absence **(B)** and presence of γ-DGG **(D)**. Solid lines: Mean of N2 (WT: 3.2 ± 0.5, *n* = 7; CR^−/−^: 3.5 ± 1, *n* = 7; WT_DGG_: 11 ± 3, *n* = 3).

To experimentally test the hypothesis of an increase in N between pulses (“overfilling” or “recruitment”), we performed MPFA of second EPSC amplitudes recorded at an ISI of 10 ms at [Ca^2+^]_e_ of 1, 2 and 10 mM (Clements and Silver, 2000). The average initial values of N (N1) in 2 mM [Ca^2+^]_e_ at unitary PF synapses were previously published to be 2.9 and 2.7 in WT and CR^−/−^, respectively (Schmidt et al., 2013). The present MPFA of second EPSC amplitudes in WT showed an increase in N (N2) to 3.2 ± 0.5 (*n* = 7) and for CR^−/−^ an increase to 3.5 ± 1 (*n* = 7; Figures 6A,B). Thus, MPFA indicates that N2 > N1. Considering that postsynaptic receptor saturation affected the amplitude of second EPSCs (see above), this analysis will even underestimate N2 as an index of release sites or RP vesicles (Scheuss et al., 2002; Silver, 2003), indicating that the increment in N2 is even larger. In order to experimentally probe this, we performed MPFA in the presence of γ-DGG (Figure 6C). EPSC amplitudes recorded from GC-to-PN pairs are small even under control conditions, making MPFA from unitary PF synapses demanding (Valera et al., 2012; Schmidt et al., 2013). On average, these amplitudes are further reduced in the presence of γ-DGG and we succeeded in performing MPFA at only three GC-PN pairs in the WT. In these experiments, we found that N2 was indeed prominently raised to a value of 11 ± 3 (Figure 6D). It is conceivable that the effects of γ-DGG deviate from linearity for low glutamate concentrations (Liu et al., 1999), which may lead to an overestimation of N in its presence, i.e., the value of 11 may be considered an upper limit for N2. Yet, these data in conjunction with the simulations (Figure 5) indicate that increased N in the second pulse will make a major contribution to PPF. Taken together, our experiments and simulations corroborate the finding that overfilling or apparent recruitment of release sites is a central mechanism of PPF at PF-to-PN synapses (Valera et al., 2012). They extend this result by showing that a transient, Ca^2+^ -dependent overfilling / recruitment mechanisms can account for the preservation of PPF at CR-deficient PF synapses.

### Coexistence of multiple forms of plasticity mechanisms

**Figure 7:**
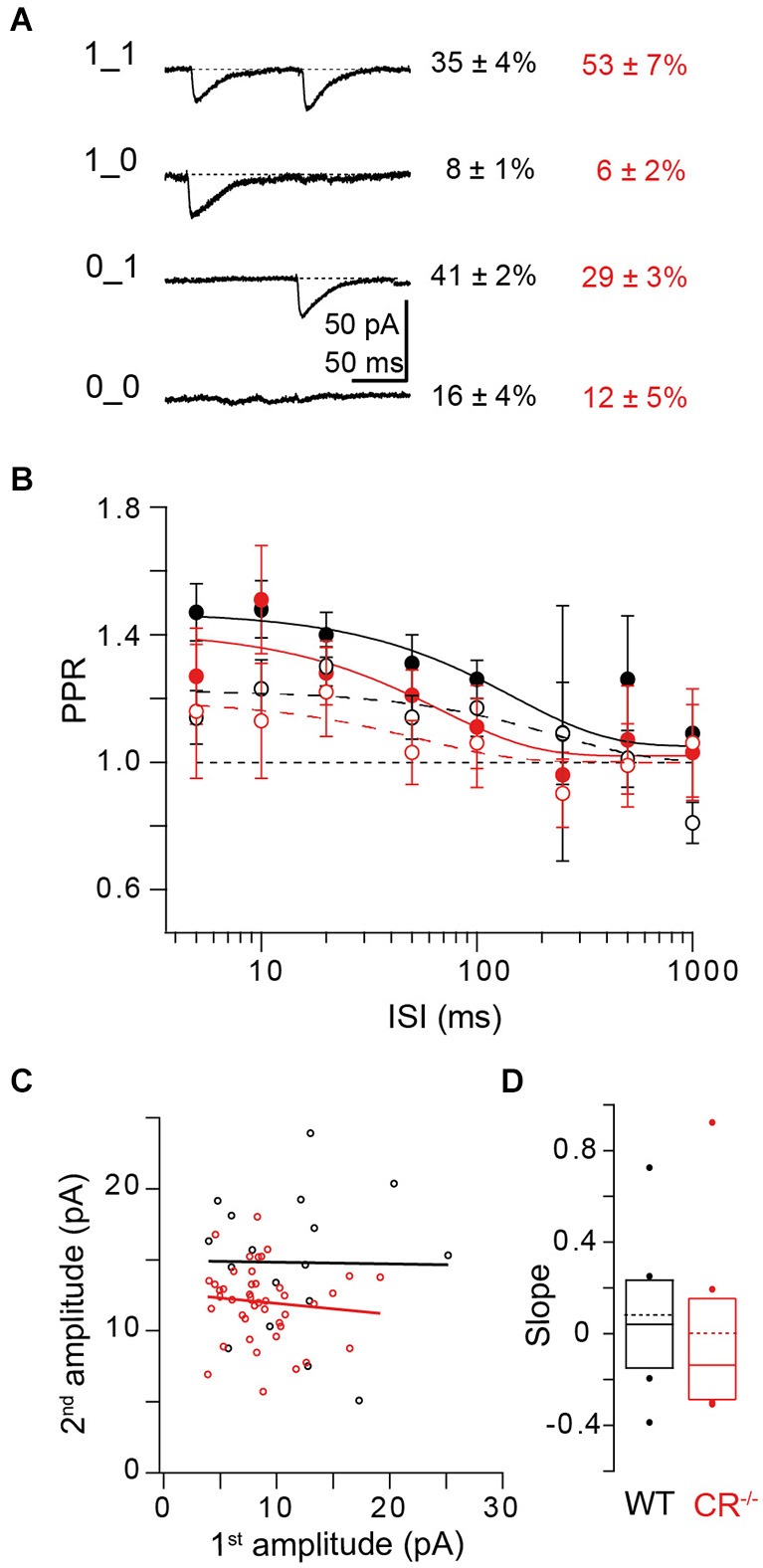
**Relationship between release and replenishment. (A)** Binary group analysis of the four possible synaptic responses in paired-pulse experiments. Left: Averaged traces from an example cell of successes on both trials (1_1), of failures on both trials (0_0) and of 1_0; 0_1 responses. Right: Percentages (mean ± SE) of the different synaptic responses in WT (black; *n* = 16) and CR^−/−^ synapses (red; *n* = 12). **(B)** ISI dependency of PPR_1_**1**/**1**_1_ (open circles and dashed lines representing exponential fits; mean ± SE) in WT (black; *n* = 12; *χ*^2^ = 4.603) and CR^−/−^ synapses (red; *n* = 12; *χ*^2^ = 0.272). Note that in both genotypes the amplitude of the second EPSC is increased at short ISI rather than depressed. The solid circles and lines show the frequency dependence of PPR_0_**1**/**1**_x_ for WT (black; *χ*^2^ = 0.32) and CR^−/−^ (red; *χ*^2^ = 0.378). Lines represent exponential fits to the data. **(C)** Second EPSC amplitudes of 1_1 events at ISI 10 ms plotted against their corresponding first amplitudes for an individual WT (black) or CR^−/−^ (red) synapse. Lines indicate linear fits to the data (black: slope = −0.01, Pearson’s R (Pr) = −0.013, *r*^2^ = 0.0002; red: slope = −0.08, Pr = −0.1, *r*^2^ = 0.01). **(D)** Summary of all slopes obtained from plots as those shown in **(C)**. Solid lines and boxes indicate median and IQR (see text), dots outlier, dashed lines the mean values for WT (black; 0.08 ± 0.11, mean ± SE; *n* = 8) and CR^−/−^ (red; 0 ± 0.14; *n* = 8; *P* = 0.442; Mann-Whitney rank sum test).

So far we focused on the synaptic responses obtained from averaging over several paired-pulse trials at a given synapse. However, individual synaptic responses to a pair of invading APs may be composed of differential sequences of successes and failures. Paired recordings allow obtaining deeper insights into the composition of individual paired-pulse trials and their relative contributions to PPF in WT and CR^−/−^. We performed a binary group analysis of the four possible synaptic responses in a paired pulse experiment, which consist of double successes (“1_1”), a success followed by a failure (“1_0”), a failure followed by a success (“0_1”), or double failures (“0_0”). The percentage of each of these response types in WT and CR^−/−^ is given in Figure 7A. In the WT 0_1 responses were recorded most frequently (41%), while in CR^−/−^ the contribution of 1_1 responses (53%) was highest. Thus, there is a clear shift towards double successes in the mutants, which is consistent with increased release probability and accelerated re- and overfilling of N.

We next focused on the 1_1 responses and calculated PPRs of individual trials by making the ratio of their second to their first EPSC amplitude. PPRs of these responses result from the combined action of presynaptic depletion and postsynaptic effects on the one hand and presynaptic facilitation mechanisms like active Ca^2+^ and overfilling on the other hand. Thus, they are an index for the relative dominance of either depression or facilitation mechanisms. At ISIs ≤100 ms PPR_1_**1**/**1**_1_ (response types used for calculating the ratio in bold) was ~1.2 in the WT, dropping to unity at larger ISI (Figure 7B open circles). PPR at short ISI was significantly larger than PPR of a hypothetical distribution of PPRs scattering around 1 with the same SE as the real data (*P* = 0.01; Kruskal-Wallis (K-W) ANOVA). In CR^−/−^ PPR_1_**1**/**1**_1_ was not significantly smaller than in WT (*P* = 0.64; K-W ANOVA). This shows that in both genotypes PPF does not only result from a higher failure rate in the first response (F1) but that the amplitude of the second response itself is facilitated rather than depressed. Since PF terminals harbor a single AZ only, this finding can only be explained if at a single AZ more than one vesicle can fuse per AP. This corroborates the notion that multi-vesicular release (MVR) can occur from PF terminals (Foster et al., 2005). It shows that the probability of MVR is increased for the second AP. Thus, second EPSC amplitudes *per se* are increased compared to the first amplitudes and contribute to PPF, both in WT and CR^−/−^.

We plotted 2nd EPSC amplitudes of double successes (1_1) against their corresponding first amplitudes (Figure 7C) and made linear fits to these data. The slopes of these fits indicate the interplay between presynaptic depletion plus postsynaptic saturation on the one hand and presynaptic replenishment on the other hand. On average (Figure 7D), the slopes of these fits were close to zero, both in WT (0.04 (−0.16–0.215), median and IQR) and in CR^−/−^ (−0.14 (−0.275–0.17); *P* = 0.442; Mann-Whitney rank sum test), indicating that median second EPSC amplitudes are “stabilized” at a given, on average slightly facilitated (Figure 7B), level, i.e., they appear essentially independent of the amplitude of the first EPSC, both in WT and KO.

The magnitude of this 1_**1**/**1**_1 PPF was much smaller than PPF of average responses (Figures 3B, 7B). This may not only result from F1 > F2, but in part also from a combination of presynaptic depletion and postsynaptic effects (cf. above). In order to substantiate this, we calculated PPR_0_**1**/**1**_x_ from the ratio of the second amplitude of 0_1 responses to the average of the first amplitude of 1_x (x = 0 or 1) responses for each ISI (Saviane and Silver, 2006; Bornschein et al., 2013). The 0_1 responses are neither affected by depletion, nor by desensitization since no prior release has occurred. In addition, release machineries of vesicles that were not released during the first pulse may still have been facilitated due to Ca^2+^ influx during the first AP. Indeed we found that PPR_0_**1**/**1**_x_ was larger (*P* = 0.019; K-W ANOVA) than PPR_1_**1**/**1**_1_, being ~1.5 at ISIs ≤100 ms in the WT (Figure 7B closed circles). In CR^−/−^ also PPR_0_**1**/**1**_x_ tended to be smaller than in the WT but again the difference was too small to reach the level of significance (*P* = 0.3; K-W ANOVA).

Taken together, our data indicate that multiple forms of plasticity mechanisms, including Ca^2+^-dependent overfilling/recruitment, active Ca^2+^, MVR, and postsynaptic effects coexist at the PF synapse. Consistent with a previous report (Valera et al., 2012), they suggest that overfilling/recruitment is the dominating mechanism of PPF at the PF to PN synapse and explain the persistence of robust PPF at CR-deficient synapses by the presumed Ca^2+^-dependence of this mechanism.

## Discussion

Using recordings from pairs of GCs and PNs connected via unitary PF synapses, we provide evidence that a Ca^2+^-driven mechanism that recovers and transiently increases N between pairs of synaptic activations dominates PPF at PF synapses of mutant mice lacking CR. Thereby, we resolve the apparent discrepancy between high *p*_r_ and the preservation of substantial PPF in these mutants. Our findings extend previous results from WT PF synapses (Valera et al., 2012) by estimating N in the second pulse, by explaining PPF in CR mutants, and by dissecting the contribution of other mechanisms to PPF, including active Ca^2+^ and MVR (cf. Foster et al., 2005).

Different mechanisms were proposed to generate PPF at distinct synapses (Zucker and Regehr, 2002; Regehr, 2012). Originally, it has been suggested that active Ca^2+^, i.e., a release machinery facilitated by a residue of bound Ca^2+^, causes facilitation (Katz and Miledi, 1968). In a simpler form of the “residual Ca^2+^ hypothesis” a residue of free Ca^2+^ ([Ca^2+^]_res_ ) from the first AP summates with free Ca^2+^ ([Ca^2+^]_i_) from the second AP, thereby, causing amplified release. However, it has been recognized early that [Ca^2+^]_res_ alone cannot account for facilitation (Blundon et al., 1993). In particular due to the large amplitude difference between [Ca^2+^]_res_ (typically < 1 μM) and nano- or microdomain [Ca^2+^]_i_ at the release site during the second AP (tens to hundred μM), simple Ca^2+^ summation is unlikely to be the major source of facilitation (Zucker and Regehr, 2002). Thus, at different synapses different mechanisms were suggested to underlie PPF. These include slow Ca^2+^ relaxation of the Ca^2+^-bound release sensor [i.e., a variant of the original active Ca^2+^ hypothesis (Yamada and Zucker, 1992; Bertram et al., 1996; Matveev et al., 2002; Bornschein et al., 2013)], separate high-affinity, slow off-rate sites for facilitation (Atluri and Regehr, 1996), elevated release site [Ca^2+^]_i_ during the second pulse (Geiger and Jonas, 2000; Felmy et al., 2003; Bollmann and Sakmann, 2005), buffer saturation (Neher, 1998a; Rozov et al., 2001), MVR (Foster et al., 2005), or an increase in N, resulting either from transient overfilling of the RP or recruitment of additional (reluctant) release sites (Millar et al., 2005; Sakaba, 2008; Valera et al., 2012).

The size of the release triggering Ca^2+^ ([Ca^2+^]_local_) has been estimated to be ~20 μM at PF synapses, decaying from peak to [Ca^2+^]_res_ of ~0.5 μM within ~2 ms (Schmidt et al., 2013). Assuming a fourth power relationship between Ca^2+^ and EPSC amplitude, the linear summation of [Ca^2+^]_res_ and [Ca^2+^]_local_ can only account for ≤10% facilitation. In addition, it has been estimated that [Ca^2+^]_res_ decays with an average *τ* of 42 ms under unperturbed conditions (Brenowitz and Regehr, 2007). Thus, PPF, which decayed with *τ* of 100 ms lasted much longer than the elevation in [Ca^2+^]_res_ (cf. Atluri and Regehr, 1996).

The signature of saturation of endogenous CaBs is a non-linear increase in [Ca^2+^]_i_ during repeated synaptic activations (Neher, 1998b). Similarly, non-linear summation of [Ca^2+^]_i_ would be expected if Ca^2+^ current facilitation is involved in the generation of PPF (Bollmann and Sakmann, 2005). Yet, at PF synapses Ca^2+^ sums linearly during repeated activations, both in WT and in CR^−/−^, making saturation of CR or Ca^2+^ influx facilitation unlikely to be involved in facilitation of release from PF terminals (Brenowitz and Regehr, 2007; Schmidt et al., 2013). Consequently, we show here that, consistent with the previous report (Schiffmann et al., 1999), robust PPF persisted in CR mutants.

Specifically for facilitation of release from PF terminals a facilitation sensor (Atluri and Regehr, 1996) or recruitment of additional release sites (Valera et al., 2012) were suggested. Present release sensor models are based on cooperative binding of several Ca^2+^ ions (Lou et al., 2005; Millar et al., 2005; Sun et al., 2007; Sakaba, 2008). Depending on the rate constants for Ca^2+^ binding and unbinding, conditioning pulses facilitate these release sensors in the absence of separate facilitation sensors. Thus, the facilitation sensor could be a variant of the facilitated release sensor and not necessarily distinct from the latter one. Considering this, our simulations explain PPF at PF synapses by a synthesis of the two previous suggestions, with the increase in N making the dominating contribution.

The magnitude of facilitation caused either by facilitation of the release machinery of non-released vesicles or by Ca^2+^-driven increase in N may change during postnatal synapse maturation since the active zone and the Ca^2+^ influx-release coupling undergoes substantial postnatal rearrangement at PF terminals (Baur et al., in press). This may explain why from experiments in younger rats the former process has been suggested (Atluri and Regehr, 1996) and in older mice the latter one (Valera et al., 2012).

STP is a central factor in neuronal information processing (Abbott and Regehr, 2004) with Ca^2+^-binding proteins being considered important regulators of PPF. Consistent with one previous report (Schiffmann et al., 1999), we found that lack of CR does not dramatically alter PPF of release from PF terminals. Since the mossy-fiber-GC-PF-pathway appears to be specialized for transmitting information in short, facilitating broad-bandwidths bursts of up to 1 kHz (Gall et al., 2003; Chadderton et al., 2004; Rancz et al., 2007; Valera et al., 2012; Ritzau-Jost et al., 2014; Rössert et al., 2014), this is a puzzling result. What might be the functional significance—if any—of CR for synaptic facilitation? Since lack of CR leads to a significant increase in *p*_r_ (Schmidt et al., 2013) the preservation of PPF is balanced by an increased vesicle replenishment and an increase in N. Given the enormous number of PF synapses in the mammalian brain and the energy costs of transmitter release and vesicle recycling, this preservation might be rather energy demanding. This suggests that one synaptic function of CR could be optimization of cerebellar energy consumption.

## Conflict of interest statement

The authors declare that the research was conducted in the absence of any commercial or financial relationships that could be construed as a potential conflict of interest.
